# Association between food insecurity and intimate partner violence: the role of gendered asset policies

**DOI:** 10.1136/bmjgh-2024-018322

**Published:** 2025-10-10

**Authors:** Faith Miller, Jenevieve Mannell, Laura Brown, Andrew Gibbs, Abigail Hatcher

**Affiliations:** 1 University College London, London, UK; 2 Institute for Global Health, University College London, London, UK; 3 University of Exeter, Exeter, UK; 4 Gillings School of global Public Health, UNC, Chapel Hill, North Carolina, USA

**Keywords:** Public Health, Global Health, Health policy, Mental Health & Psychiatry, Gender-Based Violence

## Abstract

**Introduction:**

Intimate partner violence (IPV) affects an estimated 27% of women globally, with consequences spanning mental, physical and societal well-being. Previous research identifies individual and relational risk factors for IPV, but less is known about wider structural factors. This study examines the association between food insecurity and IPV using nationally-representative data from 156 countries, exploring gendered asset policies as a potential mediator.

**Methods:**

We used nationally representative survey data on women’s experience of IPV (1993–2019) and the Food and Agriculture Organization data on moderate to severe food insecurity. Multilevel mixed-effects generalised linear models estimated the association between standardised variables for food insecurity and IPV, accounting for country-level clustering and adjusting for gross domestic product (GDP). Mediation analysis estimated the role of gendered asset policies (Women, Business and the Law Index Score). Sensitivity analyses lagged food insecurity by at least 4 years among a subset of 59 countries.

**Results:**

We examined data from 219 country-years representing 156 countries globally across the period of 1993–2019. After controlling for national GDP, models estimated an independent cross-sectional association between food insecurity and IPV (*b=*0.49; 95% CI 0.37 to 0.62). Gendered asset policies mediated 18.9% of the food insecurity-IPV relationship (indirect effect 0.099 (0.044–0.155); total effect 0.526 (0.422–0.631)). In lagged analysis, food insecurity was associated with higher IPV 2 years later (*b=*0.78; 95% CI 0.48 to 1.06), with a similar mediation effect (21.0%).

**Conclusion:**

This study is among the first to harness global data to demonstrate country-level effects of food insecurity on IPV, which countries enacting more equitable asset policies were able to mitigate a substantial proportion of. Future research should prospectively pinpoint how supportive gender policies and asset ownership can amplify the benefits of food security for women’s safety and longevity.

**WHAT IS ALREADY KNOWN ON THIS TOPIC**

**WHAT THIS STUDY ADDS**

- This study is among the first global analyses of the association between food insecurity and IPV. Among 156 countries, higher food insecurity was associated with more national exposure to IPV cross-sectionally, even when adjusting for a country’s economic status using gross domestic product. Using lagged data, when food insecurity rose at timepoint one, IPV was 0.8 SD higher at timepoint two (at least 4 years later), though stronger policies on asset ownership and inheritance offered women some protection from IPV related to food insecurity.

**HOW THIS STUDY MIGHT AFFECT RESEARCH, PRACTICE OR POLICY**

- Food security plays an important role in IPV irrespective of a nation’s income level, emphasising the necessity for governments to ensure equitable food distribution as a strategy for violence reduction. Additionally, by identifying gendered asset ownership as a critical policy lever, this research advocates for strengthening women’s rights to land and resources. Future research should explore how women’s engagement with asset ownership affects their agency and the broader impacts on food security and IPV dynamics.

## Introduction

 Intimate partner violence (IPV) is defined as behaviours by a current or previous partner that cause physical, sexual or psychological harm.^1^ Estimates from the WHO suggest that 27% of women have experienced IPV in their lifetime, often starting early in life, with around one in four ever-partnered adolescents experiencing IPV.^1^ Beyond the immediate harm to women experiencing violence, IPV contributes to a range of longer term health problems, including chronic pain, metabolic diseases and mental health disorders.^2^ IPV also has broader behavioural and social impacts, compounded by systemic barriers to escaping IPV, accessing support and seeking justice.^3^

A significant body of evidence has explored the drivers of IPV, highlighting significant overlap between the predictors of perpetration and victimisation at the individual and relationship levels.^4 5^ Predictors of perpetration and victimisation include previous abuse experiences, family conflicts, antisocial behaviours including alcohol and drug use, poor quality of friendships and romantic relationships and low socioeconomic status.^4^ In addition to these individual and relational factors, broader sociocultural and political influences are recognised as critical drivers, evidenced by the wide range in country-level estimates for the prevalence of IPV (10%–53%).^1 6^ At a contextual level, poverty is widely considered an important driver of IPV, and feminist scholarship underscores the prevalence of IPV in settings characterised by high gender inequality and restrictive gender norms.^6 7^ However, gaps in understanding these contextual drivers hinder the creation of effective, comprehensive strategies to address IPV across diverse global contexts.^6^

Emerging evidence suggests that food insecurity, defined as inconsistent access to sufficient and nutritious food, may also drive IPV.^8^,^10^ Food insecurity refers to situations where people lack consistent access to sufficient and nutritious food, including both low quantity of food as well as anxiety about food preference and quality.^11^ Approximately 2.3 billion people worldwide experienced moderate to severe food insecurity in 2023.^11^ While systematic reviews indicate an association between food insecurity and increased IPV risk,^8 12^ most existing research relies on cross-sectional and non-representative samples, limiting broader applicability.

While food insecurity is increasingly recognised as a household-level stressor that may contribute to conflict and violence, less is known about how countries can intervene in the policy environment to prevent IPV by addressing food insecurity.^9^ Gendered asset policies, which govern the rights of men and women to own, inherit and control land and property, may be particularly relevant. Ownership of physical assets enables women to generate income and store wealth, protecting them from economic shocks and providing them with claims to assets following a relationship breakdown.^13^ By enhancing women’s economic security and decision-making power, policies governing women’s ownership and control over assets could buffer households against food-related shocks and reduce women’s dependence on potentially abusive partners.^14 15^ Heise and Kotsadam found that ownership laws that favour men are strongly associated with women’s experience of violence, particularly relating to land and property.^16^ Asset ownership, particularly among women, can enhance household resilience and reduce vulnerability to food insecurity.^17^ However, the potential of gendered asset policies to mediate the relationship between food insecurity and IPV remains underexplored.

This paper aims to explore the association between food insecurity and IPV using national-level data, with a specific focus on the potential mediating role of gendered asset policies. By examining how policies relating to asset ownership and control influence the relationship between food insecurity and IPV at a country level, this research seeks to inform the development of more targeted and effective policy interventions.

## Methods

### Theoretical framing

Food insecurity is theorised to impact IPV through individual-, relationship- and social-level factors.^8^ Individual-level factors through which food insecurity may operate include stress and poor mental health, possibly leading to maladaptive coping strategies through alcohol and drug use.^918^,^21^ Relationship-level factors include food insecurity exacerbating tensions and relationship control within households, leading to higher rates of IPV.^19^,^26^ Socially, restrictive gender norms often restrict women’s access to land, credit or resources, further exacerbating IPV.^1819 24 26^,^28^ However, qualitative research implies IPV can exacerbate food insecurity by restricting survivors’/victims’ access to resources and reinforcing intrahousehold mechanisms which worsen women’s food security.^19 20 22^ This potential bidirectional relationship and the lack of evidence relating to the broader contextual drivers of IPV highlight the need for more evidence to explore the pathways between food insecurity and IPV, and potential points for intervention.^29^

### Data sources and variables

For the dependent variable in this analysis, we used data on the prevalence of IPV aggregated from 363 nationally representative surveys between 1993 and 2019. To harmonise IPV estimates across sources, we included only nationally representative surveys that used gold-standard methodologies to document self-reported experiences of physical and/or sexual violence in the past 12 months among women aged 15–49.^30^ Data were then aggregated at the country-year level to ensure consistency across time and settings. Data sources included: WHO 2018 estimates on violence against women, Demographic and Health Surveys, Reproductive Health Surveys, Multiple Indicator Cluster Surveys, United Nations Multi-country study on Women’s Health and Domestic Violence against Women, United Nations Multi-Country study of Men and Violence, International Violence Against Women Surveys, UN Women Global Database on Violence against Women and the Pan American Health Organisation’s 2018 publication on Intimate Partner Violence against Women in the Americas.

For the independent variable, we used data on food insecurity from the Food and Agriculture Organization which determines moderate or severe food insecurity (MSFI) using the Food Insecurity Experience Scale, an indicator based on eight questions regarding people’s access to adequate food, and is averaged over 3 years for each country.^31^

**Figure 1:**
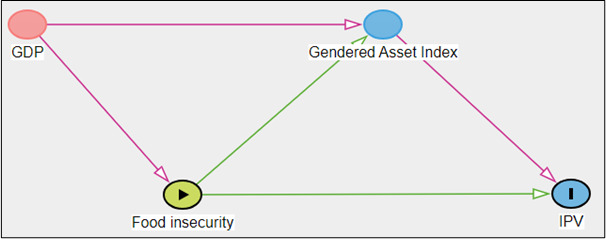
Path model presenting the hypothesised relationship between gross domestic product (GDP), foodinsecurity, gendered asset index, and intimate partner violence (IPV)Note: Independent variable (exposure), dependent variable (outcome), potential mediatingvariable, confounding variable; GDP; gross domestic product, IPV; intimate partner violence

To explore the relationships between living standards, food insecurity, gendered asset index and IPV, a path model was constructed(figure 1) . Living standards were identified as a confounding variable, and gross domestic product (GDP) based on purchasing power parity (GDP-PPP; the market value of goods and services produced in each country) was included in models to represent this.^32^ We selected GDP-PPP as it reflects a country’s relative capacity to purchase goods, including food. Given its correlation with other socioeconomic indicators, we chose GDP-PPP as a parsimonious proxy for overall living standards.

The Women, Business and the Law (WBLI) assets indicator represents the legal frameworks determining women’s rights to immovable assets, through property, land rights and inheritance.^33^ This is determined by surveying over 2400 experts and analysing domestic laws to determine gender equality in property ownership, inheritance for children and spouses, and non-monetary contributions (table 1). We selected the WBLI index over other ownership indicators because it captures the formal legal recognition of women’s rights to property and inheritance across diverse national contexts, offering a clearer reflection of institutional frameworks that can be targeted through policy interventions. However, it does not reflect women’s awareness or practical use of these rights, which are more difficult to address by national government action.^33^ Data on all variables from different sources were merged prior to the analysis (table 1).

**Table 1:** List of indicators included

Indicator	Definition and source
Food insecurity (independent variable)	Moderate or severe food insecurity (MSFI). Prevalence of moderate or severe food insecurity in the total adult population (%) (3-year average). Source: Food and Agriculture Organisation (FAO) of the UN^31^
Intimate partner violence (IPV) (dependent variable)	Women’s self-reported experience of physical and/or sexual violence in past 12 m (%).^30^ Sources: WHO VAW 2018 estimates, DHS, RHS, MICS, UN Women Global Database on Violence Against Women and Girls (VAWG), IVAWS, WHO multi-country study, PAHO 2018 publication IPV against Women in the Americas, UNMCS
Gross domestic product (GDP) (confounding)	GDP purchasing power parity (PPP) GDP based on PPP; current international $, continuous, log transformed. Source: World Bank^32^
Gendered asset index (potential mediator)	Women, Business and the Law assets sub-index score (four-category variable), determined by surveying over 2,400 experts and analysing domestic laws and regulation, is comprised of indicators on the following questions: Do a woman and a man have equal administrative power and ownership rights to immovable property, including land? Do sons and daughters have equal rights to inherit assets? Do male and female surviving spouses have equal rights to inherit assets? Does the law provide for the valuation of non-monetary contributions? Responses to these questions were scaled by 100 so that each country has a score from 0 (highest inequity) to 100 (lowest inequity). Quintiles were calculated to represent a range from 1 (lowest inequity) to 5 (highest inequity). As few country-years were ranked as quintiles 4 and 5 (n=35 and 8, respectively), these were combined into one, to produce a four-category variable (1 (lowest inequity) to 4 (highest inequity)). Source: World Bank^33^

### Analysis

We first summarised data descriptively overall and comparing across country income level (highest to lowest income) and gendered asset policy categories (lowest to highest gendered asset inequity). For continuous variables, we calculated median and interquartile ranges, comparing across groups using Kruskal-Wallis tests. For categorical variables, we calculated percentages; however, statistical comparisons are not reported due to small numbers of country-years when comparing across categories.

We used multilevel mixed-effects generalised linear regression models to explore the association between food insecurity and IPV while accounting for clustering at the country level (meglm command in Stata), with and without adjustment for GDP. We accounted for the non-normal distribution of the data by using robust standard errors. We then undertook mediation analysis (sem command in Stata) to estimate the direct and indirect effects of food insecurity on IPV while accounting for gendered asset index score and adjusting for GDP. Variables were standardised for regression and mediation models, to have a mean of 0 and a SD of 1.

An assumption of this analysis is that food insecurity precedes IPV. As a sensitivity analysis to explore this assumption, we repeated the analysis among the subset of 59 countries which had data from two timepoints at least 4 years apart (MSFI and GDP at timepoint 1 and both IPV and MSFI at timepoint 2). A 4-year lag was selected to ensure that food insecurity, which is averaged over 3 years, preceded IPV, which is based on women’s reports of IPV in the past 12 months. Regression and mediation models were run on this subgroup of countries with (i) all variables at timepoint 2 and (ii) data on MSFI, GDP and asset index were from timepoint 1 and data on IPV from timepoint 2 (at least 4 years later).

All analyses were undertaken using Stata v18.0 (StataCorp).

## Results

### Cross-sectional data

IPV prevalence was higher in low- and middle-income countries compared with high-income countries and was higher in countries with middle to high gendered asset policy inequity, compared with countries with the lowest gendered asset policy inequity (table 2). Food insecurity was significantly higher among low-income countries compared with all other higher income groups. Interestingly, food insecurity was highest among countries with midlevel gendered asset policy inequality, compared with those with the lowest and highest gendered asset inequality. When examining regional differences in gendered asset policy, a higher proportion of countries in Europe and the Organisation for Economic Co-operation and Development (OECD) had more equitable asset policies, whereas countries from South Asia, the Middle East and Africa were more inequitable.

**Table 2:** Study characteristics overall and by country income and gendered asset policy category

	Overall		By country income level									By gendered asset policy category								
	Median (IQR)	Total n	High income		Upper middle income		Lower middle income		Low income		p Value	Lowest gendered asset inequity		Mid-lower gendered asset inequity		Mid-higher gendered asset inequity		Highest gendered asset inequity		p Value
	n=219	219	n=65		n=57		n=58		n=39			n=149		n=21		n=23		n=26		
IPV	0.09 (0.05–0.16)	219	0.04 (0.04–0.06)		0.09 (0.06–0.13)		0.13 (0.10–0.23)		0.20 (0.13–0.28)		<0.001	0.07 (0.04–0.11)		0.14 (0.11–0.22)		0.23 (0.09–0.33)		0.15 (0.12–0.26)		<0.001
Food insecurity*	0.27 (0.08–0.46)	219	0.07 (0.05–0.09)		0.24 (0.14–0.43)		0.40 (0.27–0.49)		0.68 (0.55–0.79)		<0.001	0.14 (0.06–0.41)		0.62 (0.43–0.81)		0.54 (0.41–0.56)		0.33 (0.30–0.49)		<0.001
GDP-PPP	25.57 (24.39–26.76)	219	26.59 (25.34–27.62)		25.79 (24.40–26.73)		24.91 (24.39–26.36)		24.28 (23.59–25.07)		<0.001	26.03 (24.49–26.88)		24.34 (23.29–26.13)		25.22 (24.18–25.70)		25.20 (24.71–26.36)		0.01
	%	n	%	n	%	n	%	n	%	n		%	n	%	n	%	n	%	n	
Gendered asset policy		219		65		57		58		39	–									
Lowest gendered asset inequity	68.00%		98.50%	64	87.70%	50	41.40%	24	28.20%	11										
Mid-lower gendered asset inequity	9.60%		0.00%	0	3.50%	†	15.50%	9	25.60%	10										
Mid-higher gendered asset inequity	10.50%		1.50%	*	1.80%	*	22.40%	13	20.50%	8										
Highest gendered asset inequity	11.90%		0.00%	0	7.00%	4	20.70%	12	25.60%	10										
Region		219		65		57		58		39	–		149		21		23		26	–
East Asia and Pacific	11.00%		1.50%	*	8.80%	5	31.00%	18	0.00%	0		9.40%	14	9.50%	†	30.40%	7	3.80%	*	
Europe and Central Asia	11.90%		9.20%	6	28.10%	16	6.90%	4	0.00%	0		17.40%	0	0.00%	0	0.00%	0	0.00%	0	
High income: OECD	24.20%		81.50%	53	0.00%	0	0.00%	0	0.00%	0		34.90%	52	0.00%	0	4.30%	*	0.00%	0	
Latin America and Caribbean	16.00%		3.10%	†	45.60%	26	6.90%	4	7.70%	3		21.50%	32	14.30%	3	0.00%	0	0.00%	0	
Middle East and North Africa	5.50%		4.60%	3	5.30%	3	10.30%	6	0.00%	0		2.00%	3	0.00%	0	0.00%	0	34.60%	9	
South Asia	4.60%		0.00%	0	3.50%	†	5.20%	3	12.80%	5		0.00%	0	9.50%	†	0.00%	0	30.80%	8	
Central, Eastern, Southern and Western Africa*†*	26.90%		0.00%	0	8.80%	5	39.70%	23	79.50%	31		14.80%	22	66.70%	14	65.20%	15	30.80%	8	
Income group		219											149		21		23		26	–
High income	29.70%											43.00%	64	0.00%	0	4.30%	*	0.00%	0	
Upper middle income	26.00%											33.60%	50	9.50%	†	4.30%	*	15.40%	4	
Lower middle income	26.50%											16.10%	24	42.90%	9	56.50%	13	46.20%	12	
Low income	17.80%											7.40%	11	47.60%	10	34.80%	8	38.50%	10	

* Food insecurity determined using moderate or severe food insecurity (MSFI).

† We use the term ‘Central, Eastern, Southern and Western Africa’ rather than ‘Sub-Saharan Africa’ to avoid reinforcing problematic geopolitical constructs associated with that term.

GDP-PPP, gross domestic product based on purchasing power parity; IPV, intimate partner violence; OECD, Organisation for Economic Co-operation and Development.

**Figure 2:**
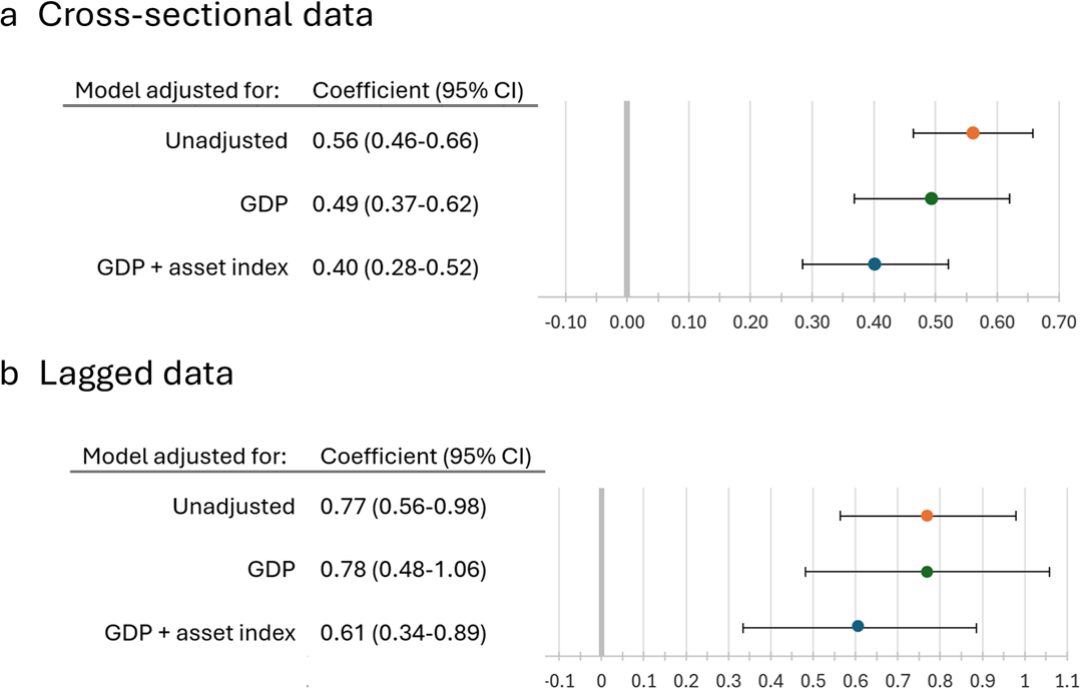
Association between food insecurity and intimate partner violence (IPV) in unadjusted and adjusted models, including a model adjusting for gendered asset index as a variable. (a) Cross-sectional model: all 219 country-years. b) Lagged model: 59 country-years with multiple timepoints at least 4 years apart. Data on food insecurity and asset index from timepoint 1 and data on IPV from timepoint 2 (at least 4 years later). When included, GDP is baseline from at timepoint 1. Note: asset index; WBLI (Women, Business and the Law) gendered assets sub-index score; gross domestic product (GDP) based on purchasing power parity (PPP).

The unadjusted model shows a significant positive association between food insecurity and IPV, with a standardised coefficient of 0.56 (95% CI 0.46 to 0.66), which attenuates slightly to 0.49 (95% CI 0.37 to 0.62) when adjusting for GDP (figure 2a). When also adjusting for gendered asset policy score, the association between food insecurity and IPV further reduces to 0.40 (95% CI 0.28 to 0.52). This highlights that GDP and gendered asset policies both account for some of the association between food insecurity and IPV.

### Lagged data

A higher proportion of high-income OECD group countries and a lower proportion of Latin American or East Asian and Pacific countries were represented in the 59 countries with lagged data (two timepoints at least 4 years apart; country characteristics in online supplemental table 1). When exploring the association between food insecurity and IPV, the association using lagged data was stronger than that in the non-lagged models, with an unadjusted standardised coefficient of 0.77 (95% CI 0.56 to 0.98; figure 2b). This association did not change when adjusting for GDP, but reduced slightly to 0.61 when adjusting for gendered asset policy score (95% CI 0.34 to 0.89; figure 2b).

### Mediation analysis

**Figure 3:**
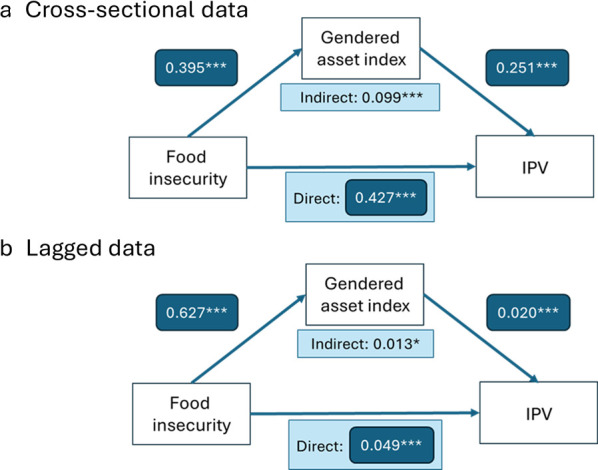
Path diagrams representing coefficients for direct and indirect effects of gendered asset index as a mediator between food insecurity and intimate partner violence (IPV). (a) Cross-sectional model: all 219 country-years. (b) Lagged model: 59 country-years with multiple timepoints at least 4 years apart. Data on food insecurity and asset index from timepoint 1, and data on IPV from timepoint 2 (at least 4 years later). Model controls for baseline GDP at timepoint 1. Note: path coefficients in dark blue box. Light blue box represents direct and indirect paths. *Significant association at p<0.05 level, ***significant association at p<0.001 level.

Mediation analysis of the cross-sectional data (219 country-years) estimated that 18.9% of the total effect was mediated by gendered asset policy (indirect effect 0.099 (95%CI 0.044 to 0.155), total effect 0.526 (95% CI 0.422 to 0.631); figure 3 and online supplemental table 2a). When running mediation models on the lagged data (59 country-years), an estimated 21.0% of the total effect was mediated by gendered asset score, even though the effect size was much smaller (indirect effect 0.013 (95% CI 0.001 to 0.024; p=0.030), total effect 0.061 (95% CI 0.038 to 0.084); figure 3 and online supplemental table 2b).

## Discussion

To our knowledge, this study represents among the first country-level global analysis of food insecurity and IPV. Our findings highlight a significant association between these conditions, adjusting for GDP, and a lagged association between food insecurity and later violence at a national level. Our mediation analysis shows that around one fifth of the total effect of food insecurity on IPV is mediated by inequity in policies governing the ownership and inheritance of assets such as property and land, highlighting a potential point for intervention.

Poverty is frequently cited as a key driver of violence at both individual and relational levels, as partners may resort to violence in response to conflict and stress over hunger.^6^ Previous work exploring the association between poverty and IPV at an individual or relational level has demonstrated mixed results,^34^ with some studies reporting higher rates of violence among wealthier women,^35^ while others have found that women’s employment and financial contribution can increase risk of IPV.^36 37^ This underscores the need for nuance when examining associations between poverty and IPV across diverse environments.

Food insecurity is a particular manifestation of poverty which can result in violence due to conflict over access to and control over resources, in addition to more general stress over finances.^38^ Our findings demonstrate that an increase in food insecurity is associated with greater country-level estimates of violence, regardless of a nation’s income level, supporting the findings of previous research that was conducted in single settings.^8 38^ Findings from our lagged models support the theory that food security has a longitudinal association with IPV at a country level, in line with longitudinal analysis of individual participant data from South Africa.^9^ These findings support the growing evidence that governments have a responsibility to ensure equitable food distribution, not only to combat hunger and poverty, but also as a potential strategy to prevent and reduce IPV.

Our findings highlight a critical policy pathway: frameworks governing the equitable ownership and inheritance of assets appear to influence the relationship between food insecurity and IPV at national levels. Strengthening legal protections for women’s land and asset ownership could enhance their decision-making autonomy and bargaining power, potentially reducing vulnerability to IPV^39^ even amid food insecurity challenges. This aligns with evidence from Kerala, India, where women’s property ownership was associated with a lower risk of IPV.^40^ Policies that strengthen women’s control over assets may include those addressing joint land registration, equal inheritance rights, financial resources (eg, tax exemptions) for women’s land ownership, protections under customary law and quotas for women’s participation in land governance, consistent with SDG 5.a.^41 42^ For example, Nepal’s implementation of tax exemptions for women’s land registration, with additional provisions for widows and single women, demonstrates how targeted asset-access policies can enhance women’s empowerment at the household level,^43^ potentially interrupting the food insecurity-IPV pathway we have identified.

A growing body of evidence shows that securing women’s property and land rights delivers multiple societal benefits through poverty reduction, environmental sustainability and smarter resource investments.^17 44^ Within agricultural practices specifically, policies aimed at improving women’s access to productive resources enable their adoption of new technologies and inclusion in agricultural support mechanisms, directly improving productivity.^45^ When women gain access to land, technologies, credit and producer networks, their economic empowerment is strengthened, potentially shielding them from violence, particularly in contexts where their economic contributions are valued.^17^ Improved access to land and assets may also result in more efficient household resource allocation, further lowering the risk of violence.^46^ Additionally, secure land rights could empower women to adopt sustainable asset and land management practices, boosting household food security and highlighting the potential for a bi-directional relationship.^47^ Our findings underscore the importance of intersectional approaches that consider how poverty interacts with broader policy environments, as these dynamics may offer deeper insights into the root causes of IPV beyond financial stress. Future research could explore how women’s access to land and resources shapes their bargaining power and agricultural engagement in both food-secure and food-insecure contexts, further illuminating the mediating mechanisms we've observed.

Diversity in how asset ownership laws are framed, interpreted and implemented across contexts underscores the need for research to understand the practical efficacy of these policies at local levels. In Mali, for instance, while statutory law formally recognises equal inheritance rights, agricultural land continues to be governed predominantly through customary practices,^42^ creating a significant implementation gap that particularly affects rural communities. This disconnect is further complicated by patrilocal marriage practices, where women relocate to their husbands' family homes, structurally reinforcing their dependence on husband-controlled assets.^48^ These examples illustrate how formal legal frameworks may fail to transform lived realities without addressing deeper sociocultural norms. Moreover, legal ownership itself presents an incomplete picture; even women who legally own assets often lack effective control or decision-making power over them.^17^ This limitation extends to our analytical approach, as the WBLI indicator we used reflects legal frameworks but does not account for women’s awareness or practical use of these rights.^33^ Furthermore, the economic value of assets owned by men and women differs between contexts, affecting the relationship between ownership policies and empowerment outcomes.^13^ Gender-sensitive asset policies might also serve as proxies for broader societal gender norms and the recognition of women’s rights, potentially impacting both food systems and IPV rates through multiple pathways.^49^ Future research should employ mixed-methods approaches to examine how women engage with asset ownership policies within specific sociocultural contexts, moving beyond legal indicators to capture lived experiences.

This study benefits from a global dataset curated from existing country-level, nationally representative surveys. A large portion of the existing research on the association between food insecurity and IPV has focused on regions with high prevalence rates, such as Africa and South Asia, or on contexts with limited social safety nets, such as the USA.^12^ By leveraging a comprehensive dataset, we reveal patterns and pathways across diverse contexts which may be obscured in regional studies.^8^ Furthermore, our sensitivity analysis using lagged data strengthens the credibility of the longitudinal assumptions, bolstering the robustness of our conclusions and highlighting the importance of exploring these relationships over time.

However, there are limitations worth noting. First, our analysis focuses on adult women’s reporting of IPV victimisation, which limits the exploration of perpetration dynamics or other individuals who are exposed to partner violence. Second, by analysing data at a country level, we were not able to explore associations between food insecurity and IPV in high-risk, marginalised communities or economically strained regions within countries.^50^ Third, use of the global WBLI asset indicator overlooks the nuance relating to policies being sensitive to local cultures and contexts which influence gender relations.^45^ Furthermore, collapsing the top two quintiles of the WBLI asset indicator into a single category, due to the small number of country-years in these groups, may have also limited our ability to distinguish between countries with moderately high levels of gender equity. Fourth, although GDP-PPP was used as a proxy for national living standards and as an indicator of potential food purchasing power, selected to maintain model parsimony, this approach may not capture the full complexity of structural drivers of IPV. Future analyses using other relevant socioeconomic indicators, such as national employment rates and education levels, which could offer additional insight into structural drivers of IPV, are needed. Finally, the food insecurity indicator was only available as a 3-year rolling average, which may have obscured short-term fluctuations in food insecurity.

### Future research directions

While this study used data at the country level, food insecurity persists among vulnerable groups even in high-income countries, such as single-parent families, low-income households, Indigenous people, refugees, migrants and those with disabilities.^51^ Future research should examine how the intersection of food insecurity, violence and women’s engagement with asset ownership policies plays out within marginalised populations across diverse cultural and policy contexts. Given that policies are implemented and experienced differently across settings, localised studies implemented mixed-methods approaches are critical to capture policy nuance and assess the practical implications of asset-based interventions. Furthermore, given the lack of understanding of women’s knowledge of or experience engaging with asset ownership policies, future work elucidating how asset ownership impacts their experiences in both food-secure and food-insecure environments would deepen our understanding of the best approaches for fostering food security and protecting women from violence. The potential for a bidirectional relationship between food insecurity and IPV emphasises the need for more longitudinal research on these relationships over time. Finally, analysis on data on violence perpetration, in addition to victimisation, would also strengthen the evidence base on associations between food insecurity and violence.

## Conclusion

This study is among the first to explore country-level associations between food insecurity and IPV. Our findings suggest that food insecurity has a measurable effect on women’s safety in relationships, and that policy frameworks governing asset ownership and inheritance could be leveraged to intervene in this association. We highlight the critical need for integrated policy and programme approaches that address both food insecurity and IPV. Specifically, our analysis demonstrates that strengthening legal frameworks governing women’s access to land and inheritance rights could potentially interrupt pathways between food insecurity and violence. By addressing these structural determinants in tandem rather than as isolated issues, policymakers may more effectively protect women’s safety while enhancing food security outcomes across diverse national contexts.

## Supplementary material

10.1136/bmjgh-2024-018322 online supplemental file 1

## Data Availability

Data are available upon reasonable request.
