# Supplementary material for: Association between food insecurity and intimate partner violence: the role of gendered asset policies
Source: BMJ Glob Health. 2025 Oct 10;10(10):e018322. doi: 10.1136/bmjgh-2024-018322 (PMC12517009; doi:10.1136/bmjgh-2024-018322)
Supplement: online supplemental file 1 [file bmjgh-10-10-s001.docx]

#### Supplemental Table 1. Characteristics of the 59 countries with multiple timepoints at least four years apart

|  | Median (IQR)  or % (by column) |
| --- | --- |
|  | N=59 |
| MSFI | 0.14 (0.06-0.47) |
| IPV | 0.07 (0.04-0.14) |
| GDP-PPP | 25.57 (24.74-26.80) |
| Gendered asset policy |  |
| Lowest gendered asset inequity | 69.49% |
| Mid-lower gendered asset inequity | 11.86% |
| Mid-higher gendered asset inequity | 10.17% |
| Highest gendered asset inequity | 8.47% |
| Region |  |
| East Asia & Pacific | 8.47% |
| Europe & Central Asia | 11.86% |
| High income: OECD | 33.90% |
| Latin America & Caribbean | 8.47% |
| Middle East & North Africa | 6.78% |
| South Asia | 3.39% |
| Central, Eastern, Southern, and Western Africa^1^ | 27.12% |
| Income group |  |
| High income | 42.37% |
| Upper middle income | 13.56% |
| Lower middle income | 25.42% |
| Low income | 18.64% |

*^1^We use the term ‘Central, Eastern, Southern, and Western Africa’ rather than ‘sub-Saharan Africa’ to avoid reinforcing problematic geopolitical constructs associated with that term*

#### Supplemental Table 2. Indirect, direct and total effects of gendered asset index score in the association between food insecurity and violence, with bootstrapped standard errors

1. Cross-sectional model: All 219 country-years

| Path effect | Coefficient | SE | z | P>z | Low 95CI | High 95CI |
| --- | --- | --- | --- | --- | --- | --- |
| Gendered Asset | | | | | | |
| Indirect | 0.099 | 0.028 | 3.510 | <0.001 | 0.044 | 0.155 |
| Direct | 0.427 | 0.051 | 8.420 | <0.001 | 0.328 | 0.527 |
| Total | 0.526 | 0.053 | 9.860 | <0.001 | 0.422 | 0.631 |

1. Lagged model: 59 country-years with multiple timepoints at least four years apart. Data on MSFI, GDP and asset index from timepoint 1, and data on IPV from timepoint 2

| Path effect | Coefficient | SE | z | P>z | Low 95CI | High 95CI |
| --- | --- | --- | --- | --- | --- | --- |
| Gendered Asset | | | | | | |
| Indirect | 0.013 | 0.006 | 2.170 | 0.030 | 0.001 | 0.024 |
| Direct | 0.049 | 0.011 | 4.270 | 0.000 | 0.026 | 0.071 |
| Total | 0.061 | 0.012 | 5.250 | 0.000 | 0.038 | 0.084 |
